# Predictive value of [^99m^Tc]Tc-MAA SPECT/CT personalised dosimetry in ^90^Y radioembolization of hepatocellular carcinoma: a DOSISPHERE-01 ancillary study

**DOI:** 10.1007/s00259-026-08079-8

**Published:** 2026-08-11

**Authors:** Grace Keane, J Chalaye, B Guiu, L Tselikas, K. D Fowers, E Boucher, E Garin, X Palard, M. G. E. H. Lam

**Affiliations:** 1 Department of Radiology and Nuclear Medicine, University Medical Center, Utrecht, 3508 GA The Netherlands; 2 Assistance Publique Hôpitaux de Paris, Paris, France; 3 Department of Radiology, St-Eloi University Hospital, Montpellier, France; 4 Département d’Anesthésie Chirurgie et Interventionnel, Interventional Radiology unit, Gustave Roussy Université Paris Saclay, Villejuif, France; 5 Boston Scientific Corporation, Marlborough, MA USA; 6 Department of Nuclear Medicine, Centre de Lutte Contre le Cancer Eugène Marquis, Rennes, France

**Keywords:** Radioembolization, Hepatocellular Carcinoma, Technetium-99m macroaggregated albumin, SPECT/CT, PET/CT, Yttrium-90

## Abstract

**Purpose:**

The DOSISPHERE-01 study demonstrated a survival benefit for patients treated with ^90^Y radioembolization using personalised [^99m^Tc]Tc-MAA guided versus standard dosimetry guided treatment. This ancillary analysis complements DOSISPHERE-01 by reporting post-treatment dosimetry and assessing agreement between predictive ([^99m^Tc]Tc-MAA) and post-treatment (^90^Y) absorbed doses.

**Materials and methods:**

All DOSISPHERE-01 patients with both pre-treatment [^99m^Tc]Tc-MAA SPECT/CT and post-treatment ^90^Y imaging were eligible. Multicompartment dosimetry was performed using two segmentation methods: anatomic (MRI/CT) and count-threshold based (SPECT). Mean normal liver tissue and index-lesion absorbed dose, and dose volume histogram (DVH) metrics were determined using Simplicit^90^Y (Mirada Medical, Oxford UK). Agreement between [^99m^Tc]Tc-MAA and ^90^Y absorbed doses was assessed via Bland-Altman and paired-sample analysis, with correlation assessed via linear-regression.

**Results:**

Thirty-seven patients were included: 19 from the personalised arm and 18 from the standard dosimetry arm. Pre- and post-treatment absorbed dose differences were not statistically significant for the index lesion (241 Gy vs. 229 Gy, *p* = 0.15; mean difference 11.9 Gy; 95% LoA − 84.3 to 108.2 Gy), Perfused Volume Normal Liver Tissue (PVNT) (91 Gy vs. 99 Gy, *p* = 0.14; mean difference − 8.2 Gy; 95% LoA − 71.6 to 55.1 Gy), or DVH metrics (V200, D70, D50) using anatomic segmentation. Corresponding metrics were significantly different using count-threshold segmentation (*p* < 0.05). For both methods, pre- and post-treatment mean index lesion and PVNT absorbed doses, and V200, D70, D50 were strongly correlated (Pearsons Correlation Coefficient > 0.65, *p* < 0.05).

**Conclusion:**

^90^Y absorbed dose outcomes in the DOSISPHERE-01 study corresponded closely with [^99m^Tc]Tc-MAA predictive dosimetry, confirming the validity of [^99m^Tc]Tc-MAA for dosimetry-based treatment planning in this clinical scenario. Agreement was maximised using anatomic segmentation.

**Supplementary Information:**

The online version contains supplementary material available at https://doi.org/10.1007/s00259-026-08079-8.

## Introduction

Primary liver cancer, of which hepatocellular carcinoma (HCC) is the most prevalent type, ranks as the second leading cause of cancer-related death worldwide [1]. Radioembolization is a well-established treatment modality for unresectable HCC [2–4] and is recognised in the Barcelona Clinic Liver Cancer (BCLC) treatment guidelines [5, 6]. This locoregional therapy involves the transarterial delivery of β-emitting yttrium-90 (^90^Y) microspheres. By selectively administering these microspheres into the hepatic artery the procedure exploits the liver’s dual blood supply – where tumours preferentially receive blood from the hepatic artery, and normal liver tissue via the portal vein – thus achieving a high tumour absorbed dose while largely sparing normal liver parenchyma.

^90^Y radioembolization is preceded by diagnostic angiography and Technetium-99 macroaggregated albumin ([^99m^Tc]Tc-MAA) SPECT/CT imaging, routinely performed to identify patients at risk of complications due to hepatopulmonary or gastro-intestinal shunting [7]. However, this procedure may also serve as the ‘scout study’ from which the intrahepatic distribution of [^99m^Tc]Tc-MAA is used to predict the tumour and normal liver tissue absorbed dose and calculate the required activity [8]. Standard single compartment dosimetry (SCD) for ^90^Y glass microspheres (TheraSphere, Boston Scientific) typically involves delivering an average absorbed dose of 120 Gy ± 10% to the treated volume [9–11]. In this approach the distribution pattern between tumour and normal liver tissue is not considered. This represents a simplification of the actual distribution as absorbed dose is escalated in hypervascular tumours relative to the surrounding normal liver tissue [12]. Several retrospective studies demonstrated the benefits of implementing pre-treatment multicompartment dosimetry (MCD) [13–15]. The MCD approach accounts for absorbed doses to multiple compartments, including tumour and normal liver tissue, ensuring the tumour attains an optimal absorbed dose while minimising exposure to normal liver tissue [16, 17]. The DOSISPHERE-01 [18] trial was the first prospective, randomised study to demonstrate that implementing personalised MCD (which ensured a tumoricidal absorbed dose threshold of at least 205 Gy, and if feasible greater than 300 Gy) significantly improved objective response rate (ORR) without increasing toxicity. Median overall survival (OS) in the intention-to-treat population was 26.6 months (95% CI, 11.7–not reached [NR]) in the personalised MCD group versus 10.7 months (95% CI, 6.0–16.8) in the standard SCD group (HR 0.421, 95% CI 0.215–0.826; *p* = 0.0096). Additionally, bridging to curative intent was achievable to a greater degree when personalised MCD was implemented (36% of patients were downstaged to resection versus 4% with standard SCD).

The significant improvements in ORR and OS achieved with personalised MCD treatment planning, together with its potential to enable treatment stage migration, highlighted the effectiveness of this approach. However, its success is dictated by the predictive capability of the [^99m^Tc]Tc-MAA distribution used to simulate the post-treatment absorbed dose distribution [19–22]. Best practice guidelines recommend the use of post-treatment imaging with ^90^Y bremsstrahlung SPECT/CT or ^90^Y PET/CT to validate the [^99m^Tc]Tc-MAA simulation, and specifically, to confirm the absorbed dose to tumour and normal liver tissue after the therapeutic procedure [23]. It is important to complement the estimated absorbed doses from [^99m^Tc]Tc-MAA dosimetry reported in DOSIPHERE-01, with the actual absorbed doses that were delivered in the ^90^Y therapeutic procedure.

Different segmentation methods have been utilised and reported on in dosimetry analysis studies [16, 24], each with inherent trade-offs. Anatomic segmentation, based on delineation from anatomic imaging co-registered to [^99m^Tc]Tc-MAA SPECT/CT, is susceptible to misregistration and interval progression. Count-threshold segmentation, derived from activity isocontours directly on [^99m^Tc]Tc-MAA SPECT/CT, reduces these errors, but variability is introduced due to operator-dependent thresholding. The relative agreement in pre- and post-treatment dosimetry as a function of segmentation methods remains inadequately defined.

This study reports the therapeutic absorbed doses delivered to patients in the DOSISPHERE-01 trial derived from an MCD analysis of post-treatment imaging. Additionally, an analysis was conducted to assess the relationship between dosimetry metrics from the [^99m^Tc]Tc-MAA pre-treatment study and the ^90^Y post-treatment study, focusing on the segmentation method (anatomic vs. count threshold) that optimises agreement between pre- and post-treatment.

## Materials and methods

### Study participants

This study analysed prospectively collected data from the DOSISPHERE-01 trial; a randomised, multicentre, open-label phase 2 study of radioembolization treatment of HCC with ^90^Y glass microspheres, conducted at four centres in France between December 2015 and January 2018. The trial adhered to the Declaration of Helsinki and received ethics committee approval. The trial protocol and main results are available elsewhere [18, 25, 26].

Subjects were eligible for the DOSISPHERE-01 trial if they had histologically confirmed HCC not amenable to surgery or local ablative treatment, with at least one measurable lesion ≥ 7 cm and a hepatic reserve ≥ 30% post-radioembolization. Main exclusion criteria included extrahepatic spread (with the exception of lymph nodes of the hilum of size < 2 cm) and contraindications to radioembolization as assessed by [^99m^Tc]Tc-MAA scintigraphy and diagnostic angiography.

The [^99m^Tc]Tc-MAA study involved administration of ca. 150 MBq ^99m^Tc-labelled macroaggregated albumin over 20–30 s, with planar imaging (for lung shunt fraction estimation) and SPECT/CT (to confirm absence of digestive shunting and intrahepatic dosimetry) acquired within one hour of injection. Diagnostic angiography facilitated arterial mapping, catheter positioning, and embolisation of gastrointestinal and arterial branches (where indicated). To maximise reproducibility, matched catheter positions (i.e., equivalent artery, distance from arterial bifurcations, and intraluminal angulation) [26] were required in the diagnostic and therapeutic angiographies. Post-treatment imaging was performed the day following treatment and could comprise either ^90^Y PET/CT or SPECT/CT. Imaging parameters are detailed in Supplementary Table 1.

In the personalised arm, dosimetry targets were ≥ 205 Gy to the tumour (with a target range exceeding 300 Gy if possible) and < 120 Gy to perfused normal liver tissue, with a 30% hepatic reserve. In the standard arm the target was 120 ± 20 Gy average to the perfused volume, regardless of tumour absorbed dose. Full details of the inclusion and exclusion criteria and required imaging studies are provided by Garin et al. [19, 26].

Participants were excluded from the current analysis if supporting dosimetric data (e.g., injected ^90^Y activity, lung shunt) were unavailable, or if a diagnostic contrast-enhanced baseline (CT or MRI) study, [^99m^Tc]Tc-MAA SPECT/CT study, or post-treatment (^90^Y bremsstrahlung SPECT/CT or ^90^Y PET/CT) study was unavailable for centralised reading. Patients lacking baseline imaging, but with all other data available, were included in a supplementary sub-analysis (Supplementary Tables 3 and Figs. 6 and 7).

### Pre- and post-treatment dosimetry

A pre- and post-treatment dosimetry analysis was conducted using Simplicit^90^Y (Mirada Medical, Oxford, UK). Dosimetry was performed using a multicompartment Medical Internal Radiation Dose (MIRD) methodology [27], employing two distinct segmentation approaches: anatomic segmentation (CT/MRI-based) and count-threshold segmentation ([^99m^Tc]Tc-MAA SPECT-based). Mean absorbed dose was calculated for volumes of interest (VOIs) including index lesion and perfused normal liver tissue. Dose volume histogram (DVH) metrics for the index lesion were extracted, including D95, D70, D50, and V200. D95, D70, and D50 represent the absorbed dose (Gy) received by at least 95%, 70%, and 50% of the index lesion volume, respectively, while V200 represents the percentage of the index lesion volume receiving an absorbed dose of at least 200 Gy.

An investigator specified lung shunt fraction (LSF) was manually entered. The LSF and lung dose were calculated from planar [^99m^Tc]Tc-MAA imaging, using the formulas specified in the EANM Standard Operational Procedures for [^99m^Tc]Tc-MAA dosimetry in liver radioembolization with ^90^Y microspheres [9]. LSF and lung dose were not reassessed post treatment from ^90^Y imaging.

Registration of the baseline imaging dataset (CT or MRI) and the [^99m^Tc]Tc-MAA SPECT/CT (pre-treatment) or ^90^Y imaging (post-treatment) was performed via rigid registration and refined using a manual adaptation. A quality check using semi-transparent overlays was performed.

### Anatomic segmentation

The whole liver volume was delineated on baseline imaging and was propagated to all registered datasets. The index lesion and portal vein thrombosis (PVT) (if applicable) were segmented on baseline images. Contrast-enhanced CT/MRI images were used for index lesion delineation and necrotic components were excluded. The perfused volume was primarily determined from baseline diagnostic imaging using vascular anatomy for boundary definition. In the absence of cone-beam CT (typically used for perfused volume segmentation), the co-registered [^99m^Tc]Tc-MAA SPECT/CT was used as a secondary adjunct to assist in identifying the edge of the volume. Tumour volume(s) (i.e., all identifiable lesions) and the PVT VOI (where applicable) were subtracted from the perfused liver volume, and the resulting VOI was defined as the perfused volume normal liver tissue (PVNT). The injected activity was adjusted for the lung shunt fraction and the residual fraction (i.e., the residual activity in the injection system not administered to the patient) to determine the activity in the liver. A patient-specific calibration (i.e., the ratio between the known activity in the liver and the counts in the perfused volume) and a local deposition model (which assumes that all energy emitted in a VOI is entirely deposited within that same VOI) were applied, and the absorbed doses for the respective VOIs were calculated using the MCD MIRD formula:

$$\:{D}_{VOI}=\:\frac{(50\:\times\:\:{A}_{liver})}{({M}_{VOI}\:\times\:\:{\sum\:C}_{i})}{\:C}_{VOI}$$

Where D_VOI_ is the absorbed dose (Gy) for a given VOI, the factor 50 (J/GBq) is the energy deposited in the region over time per activity injected for ^90^Y, A_liver_ is the activity (GBq) in the liver, M_VOI_ is the mass (kg) of the VOI, $\:\sum\:{C}_{i}$ is the total counts in the perfused volume and C_VOI_ is the counts in the VOI. Counts that appear outside of the perfused volume were not considered to be caused by activity physically outside the perfused volume. Rather these are caused by the limitations of the imaging system; noise, artefacts and partial volume effects. These were not accounted for in the patient relative calibration, and were considered to be negligible.

### Count-threshold segmentation

For count-threshold segmentation, the perfused volume, index lesion and PVT were delineated based on a reviewer-dependent count-based threshold of SPECT data. The nuclear medicine physician reviewed and edited the threshold limits, to ensure that the VOI boundaries were correct, and the volumes were not over or under-estimated. For example, in hypovascular tumours, low count-thresholds risked capturing spurious counts (i.e., noise) beyond the index lesion volume, the VOI boundary was, in such cases, constrained to the anatomic index lesion margin.

For post-treatment dosimetry analysis, the baseline image was registered to the ^90^Y SPECT/CT or ^90^Y PET/CT. Segmentations generated during the anatomic and count-threshold pre-treatment workflow were then imported and propagated to the post-treatment study without modification, and absorbed dose to the respective compartments was calculated as per the above.

### Outcome measures

The endpoints of interest included mean absorbed dose for index lesion and PVNT. Secondary endpoints included dose volume histogram metrics D95, D70, D50 and V200.

### Statistical analysis

Agreement between pre-treatment and post-treatment dosimetry outcomes was assessed using Bland–Altman and paired t-test analysis. For the Bland-Altman analysis the 95% confidence intervals were computed by multiplying the standard error of the upper and lower 95% limits of agreement by the critical value from the t-distribution with *n* − 1 degrees of freedom. The precision of these 95% limits was determined based on the number of procedures analysed, the standard deviation of the difference(s), and the degrees of freedom (*n* − 1), following the method outlined by Bland and Altman [28].

Linear regression analysis was conducted with a least mean square method. Confidence intervals were generated via percentile bootstrapping. Summary statistics included Pearson’s correlation coefficient and R^2^ values.

The mean percentage bias between the pre- and post-treatment index lesion absorbed dose and PVNT absorbed dose was calculated as follows:

$$\begin{aligned}\;Mean\:Percentage\:Bias=\frac{{\sum\:}_{n=1}^{N}\frac{\left({D}_{pre-treatment,\:n}-{D}_{post-treatment,\:n}\right)}{{D}_{post-treatment,n}}}{N}\end{aligned}$$

Where D_pre−treatment_ is the absorbed dose determined for the respective VOIs from pre-treatment [^99m^Tc]Tc-MAA dosimetry, D_post−treatment_ is the absorbed dose determined for the respective VOIs via post-treatment ^90^Y dosimetry and N is the total number of cases.

## Results

Thirty-seven participants were included in this analysis after exclusion of 19 patients; five due to unavailability of a diagnostic quality, contrast enhanced baseline (CT or MRI) study, eight due to unavailability of a [^99m^Tc]Tc-MAA SPECT/CT and six due to unavailability of post-treatment ^90^Y imaging. Of the 37 patients included, 18 were treated in the standardised SCD arm, and 19 were treated in the personalised MCD arm. Baseline imaging comprised either a contrast-enhanced CT (89%) or MRI (11%). Twenty-three patients underwent post-treatment ^90^Y PET/CT (62%) and 14 underwent ^90^Y SPECT/CT (38%). The median interval between baseline imaging and [^99m^Tc]Tc-MAA SPECT/CT was 10 days (± 11), and between baseline and post-treatment imaging 22 days (± 13). The mean prescribed activity was 3.4 GBq (± 1.5 GBq), and the mean residual fraction was 2.8% (± 5.4%). The mean lung shunt fraction was 4.2% (± 6.5%) (Table 1).

**Table 1:** Baseline and treatment characteristics

Parameter	All patients (*N* = 37)	Personalised (*N* = 19)	Standardised (*N* = 18)
Population			
Mean Age (± SD) (years)	64 ± 13	64 ± 10	64 ± 15
Gender			
Male / Female	35 (95%) / 2 (5%)	18 (95%) / 1 (5%)	17 (94%) / 1 (6%)
Liver Cirrhosis	29 (78%)	15 (79%)	14 (78%)
ECOG Status			
0	20 (54%)	9 (47%)	11 (61%)
1	17 (46%)	10 (53%)	7 (39%)
Tumour characteristics			
BCLC Status			
B	5 (14%)	3 (16%)	2 (11%)
C	32 (86%)	16 (84%)	16 (89%)
Location			
Lobar	20 (54%)	11 (58%)	9 (50%)
Sub-Lobar	15 (41%)	7 (37%)	8 (44%)
Segment 4	2 (5%)	1 (5%)	1 (6%)
Liver functional reserve test			
Child Pugh Status			
A5	28 (76%)	14 (74%)	14 (78%)
A6 or B7	9 (24%)	5 (26%)	4 (22%)
Treatment			
Activity			
Total (SD) (GBq)	3.4 (1.5)	4.2 (1.6)	2.6 (0.9)
Residual (SD) (%)	2.8 (5.4)	3.1 (7.1)	2.5 (2.3)
Injected (SD) (GBq)	3.3 (1.4)	4.0 (1.4)	2.6 (0.9)
Lung Shunt (SD) (%)	4.2 (6.5)	4.4 (7.2)	3.9 (5.6)
Lung Dose (SD) (Gy)	6.4 (8.7)	6.7 (8.1)	6.0 (9.3)
Time Interval			
Mean Baseline to Pre-treatment (SD) (Days)	10 (11)	9 (8)	12 (13)
Mean Baseline to Post-treatment (SD) (Days)	22 (13)	19 (9)	25 (15)

*SD *Standard Deviation, *ECOG *Eastern Cooperative Oncology Group, *BCLC *Barcelona Clinic Liver Cancer

**Fig. 1:**
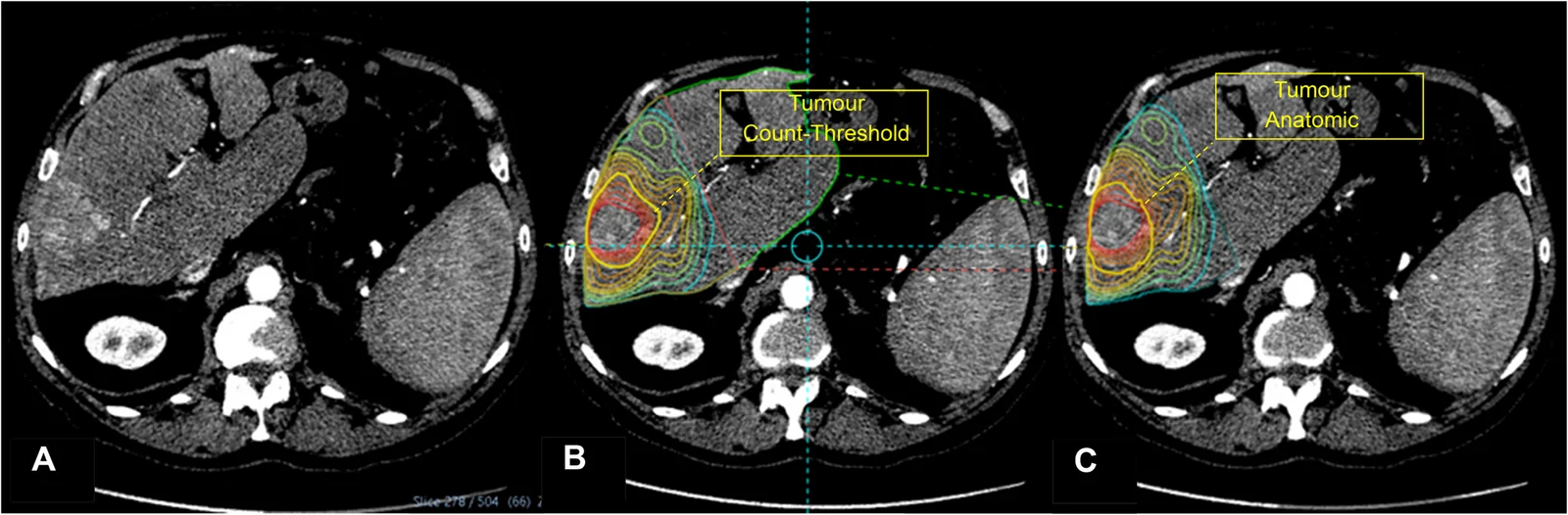
Comparison of count-threshold and anatomic segmentation methods applied to the same case, demonstrating subtle differences in tumour VOI delineation between the two approaches. Count-threshold segmentation yielded a smaller tumour volume and greater discrepancy between pre and post treatment dose measurements, whereas anatomic segmentation produced a larger tumour volume and greater concordance between corresponding dose measurements. **A **Contrast enhanced CT demonstrating a tumour (6 cm) in the right lobe. **B** Segmentation of the tumour using a count-threshold method. With this method the tumour volume was 112.8 cm3, pre-treatment tumour dose was 460.2 Gy and post-treatment tumour dose was 344.4 Gy. **C **Segmentation of the tumour using an anatomic method. With this method the tumour volume was 130.5 cm3, pre-treatment tumour dose was 382.2 Gy and post-treatment tumour dose was 362.2 Gy

The mean index lesion absorbed dose at pre- and post-treatment was 241 Gy (± 122 Gy) vs. 229 Gy (± 122 Gy) (*p* = 0.15) for anatomic segmentation and 297 Gy (± 141 Gy) vs. 246 Gy (± 128 Gy) (*p* < 0.05) for count-threshold segmentation. The corresponding mean PVNT absorbed dose was 91 Gy (± 50 Gy) vs. 99 Gy (± 47 Gy) (*p* = 0.14) for anatomic segmentation and 69 Gy (± 42 Gy) vs. 97 Gy (± 47 Gy) (*p* < 0.05) for count-threshold segmentation (Table 2). The index lesion volume for anatomic and count-threshold segmentation was 481 ml (± 327 ml) and 426 ml (± 284 ml) (*p* < 0.05) respectively. The PVNT volume for anatomic and count-threshold segmentation was 646 ml (± 381 ml) and 703 ml (± 388 ml) (*p* < 0.05). Figure 1 shows an example patient, with the index lesion segmented using both anatomic and count-threshold methods.

**Table 2:** Pre- and post-treatment dosimetry statistics for anatomic and count-threshold segmentation methods

Segmentation method	Structure	DVH Metric	Pre-treatment	Post-treatment	*P*-Value	PCC	*P*-value
Anatomic	Index lesion	Dmean (SD)	241 (122)	229 (122)	0.15	0.92	< 0.05
		D95 (SD)	57 (42)	65 (55)	0.38	0.32	0.05
		D70 (SD)	155 (89)	146 (92)	0.37	0.80	< 0.05
		D50 (SD)	220 (118)	206 (124)	0.11	0.92	< 0.05
		V200 (SD)	49 (27)	45 (29)	0.17	0.80	< 0.05
	PVNT	Dmean (SD)	91 (50)	99 (47)	0.14	0.78	< 0.05
Count-threshold	Index lesion	Dmean (SD)	297 (141)	246 (128)	< 0.05	0.87	< 0.05
		D95 (SD)	160 (92)	74 (59)	< 0.05	0.38	< 0.05
		D70 (SD)	216 (110)	161 (95)	< 0.05	0.79	< 0.05
		D50 (SD)	267 (128)	220 (122)	< 0.05	0.88	< 0.05
		V200 (SD)	62 (30)	48 (27)	< 0.05	0.66	< 0.05
	PVNT	Dmean (SD)	69 (42)	97 (47)	< 0.05	0.72	< 0.05

*SD* Standard Deviation, *PVNT* Perfused volume normal liver tissue, *PCC* Pearsons Correlation Coefficient

**Fig. 2:**
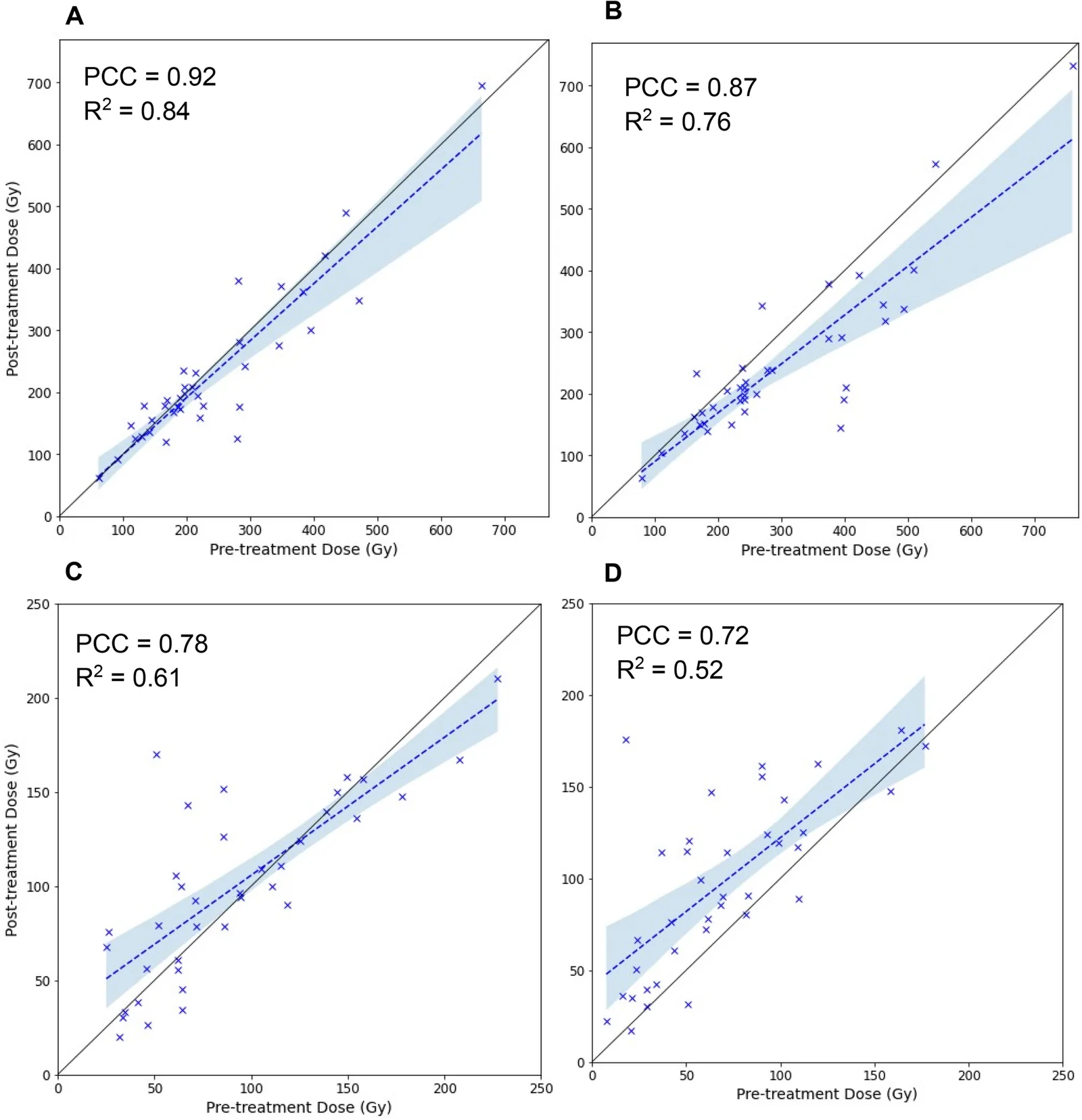
Linear regression plots for index-lesion and perfused volume normal liver tissue. Panel A and B shows index lesion pre and post-treatment correlation for anatomic (**A**) and count-threshold segmentation (**B**). Panel C and D shows PVNT pre and post-treatment correlation for anatomic (**C**) and count-threshold segmentation (**D**)

A statistically significant correlation was observed between pre- and post-treatment mean absorbed dose and dose volume histogram metrics (Table 2) for both the index lesion and PVNT. Pearson’s correlation coefficients (PCC) were 0.92 for the index lesion using anatomic segmentation and 0.87 using count-threshold segmentation. For PVNT, PCC values were 0.78 and 0.72 for anatomic and count-threshold segmentation, respectively (Fig. 2).

**Fig. 3:**
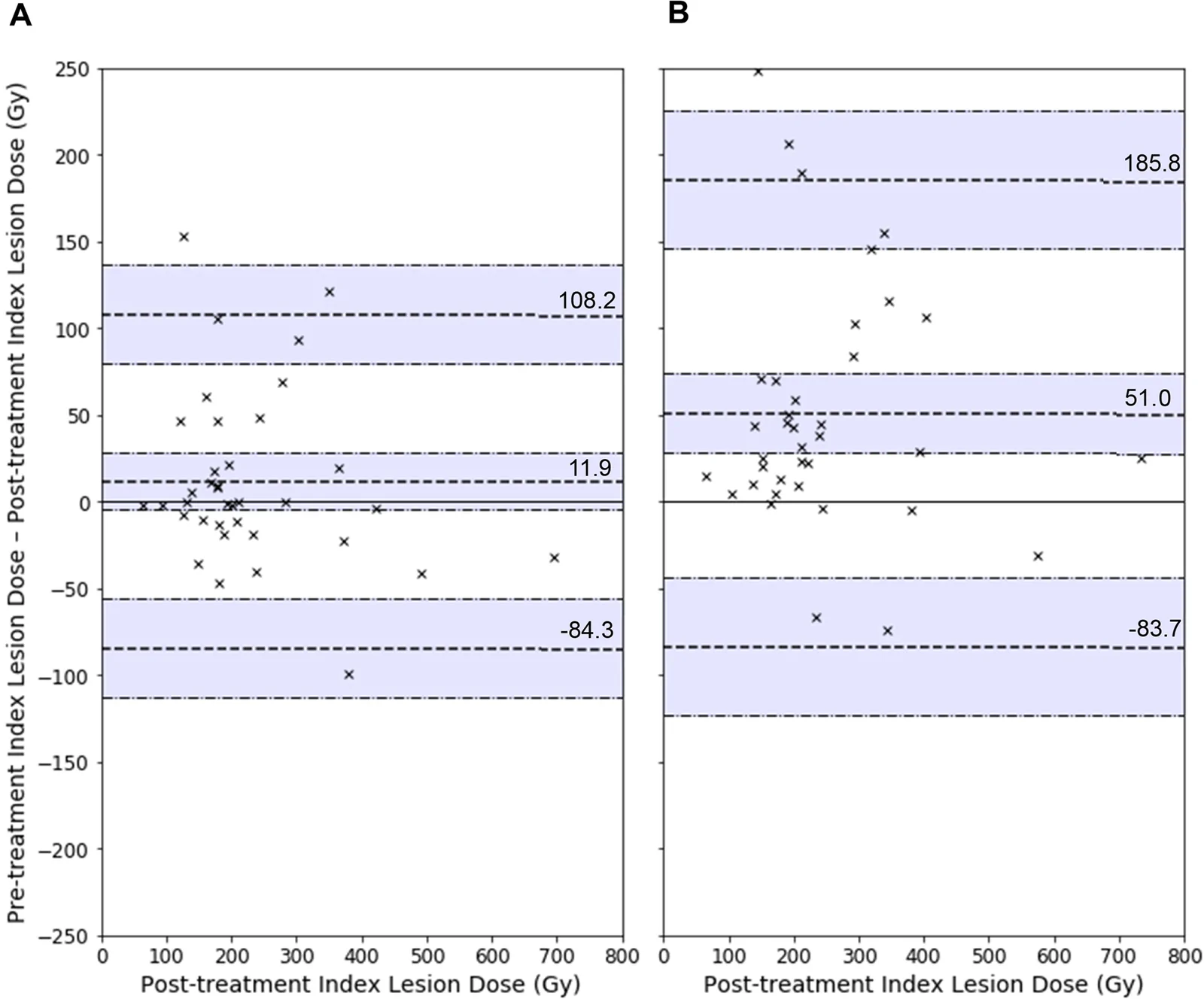
Bland-Altman plots for the index lesion analysis. The difference between [^99m^Tc]Tc-MAA and ^90^Y index lesion dose is plotted against post-treatment index lesion dose for (**A**) anatomic segmentation and (**B**) count-threshold based segmentation. The 95% limits of agreement (LoA) are indicated and surrounded by two dotted lines indicating the standard error of the 95% LoA

In both methods, pre-treatment dosimetry seemed to overestimate the index lesion absorbed dose, with a greater positive bias observed for count-threshold segmentation compared to anatomic segmentation (24.8% vs. 7.7%). The mean difference between pre- and post-treatment index lesion absorbed dose was 11.9 Gy for anatomic segmentation and 51.0 Gy for count-threshold segmentation. The 95% limits of agreement were narrower for anatomic segmentation (–84.3 to 108.2 Gy) (Fig. 3A) than for count-threshold segmentation (–83.7 to 185.8 Gy) (Fig. 3B).

**Fig. 4:**
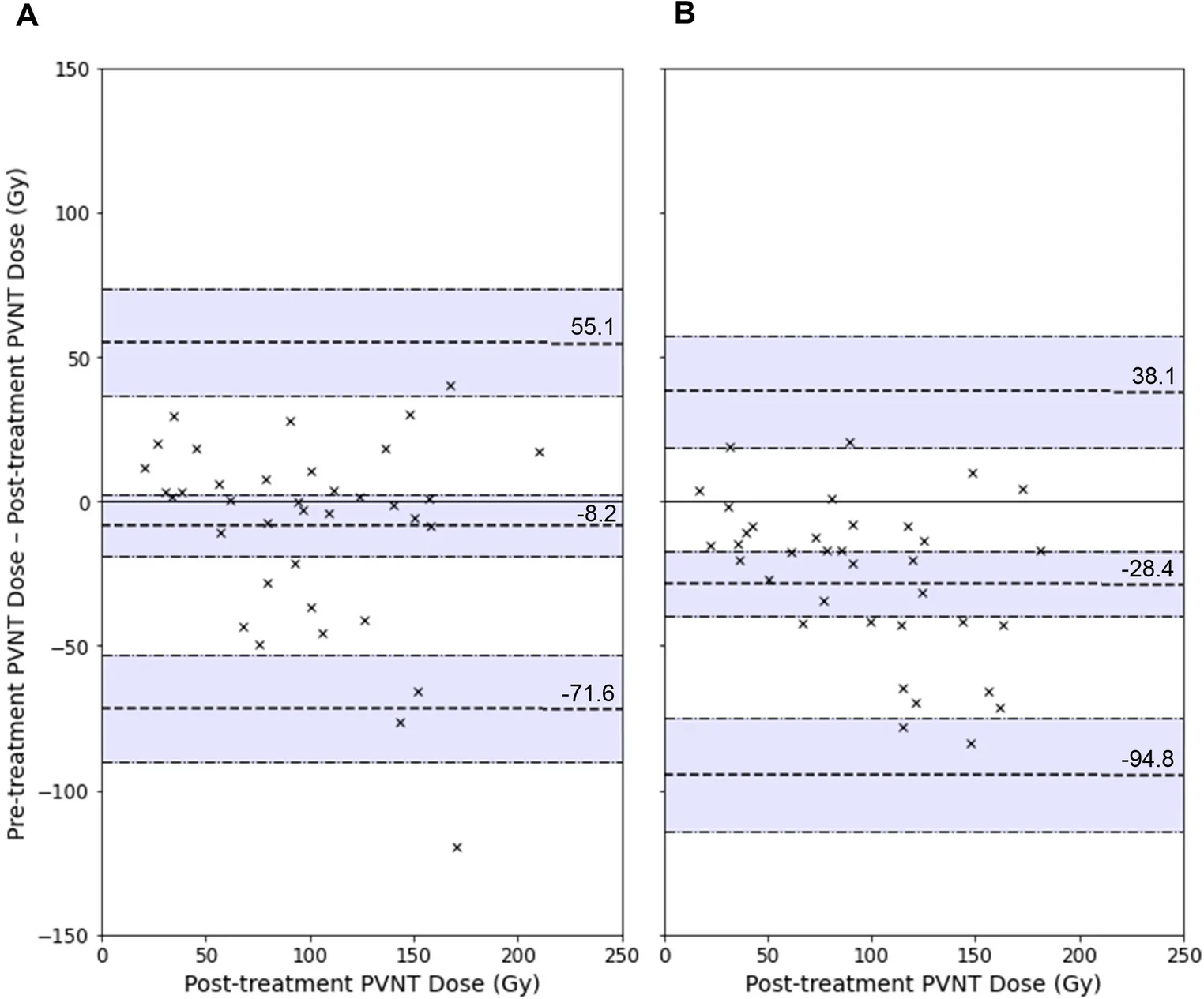
Bland-Altman plots for the perfused volume normal liver tissue (PVNT) analysis. The difference between [^99m^Tc]Tc-MAA and ^90^Y PVNT dose is plotted against post-treatment PVNT dose for (**A**) anatomic segmentation and (**B**) count-threshold based segmentation. The 95% limits of agreement (LoA) are indicated and surrounded by two dotted lines indicating the standard error of the 95% LoA

For PVNT, pre-treatment dosimetry seemed to underestimate normal liver tissue absorbed dose, with biases of − 26.7% for count-threshold segmentation and − 2.1% for anatomic segmentation. The mean difference between pre- and post-treatment PVNT absorbed dose was − 8.2 Gy with anatomic segmentation and − 28.4 Gy with count-threshold segmentation. The 95% limits of agreement for anatomic segmentation were − 71.6 to 55.1 Gy (Fig. 4A), and for count-threshold segmentation were − 94.8 to 38.1 Gy (Fig. 4B).

D70, D50, and V200, pre- and post-treatment values were strongly correlated (PCC > 0.65) for both segmentations methods, whereas D95 demonstrated weaker correlation (anatomic: PCC = 0.32; count-threshold: PCC = 0.38) (Table 2). Pre- and post-treatment differences for dose volume histograms metrics are summarised in Table 2 and Bland-Altman plots in Supplemental Figs. 1–4, with narrower limits consistently observed for anatomic segmentation. Supplemental Fig. 5 shows an example case with large discrepancy in pre- and post-treatment dose.

For the personalised MCD cohort (*n* = 19) and the standard SCD cohort (*n* = 18), Supplemental Table 2 lists the mean pre- and post-treatment index lesion and PVNT absorbed doses. In both MCD and SCD subgroups, anatomic segmentation showed no significant pre- to post-treatment absorbed dose changes, whereas count-threshold segmentation demonstrated significant differences.

DOSISPHERE-01 demonstrated a significant difference in the proportion of patients achieving the minimum tumour absorbed dose objective of 205 Gy between the personalised MCD and standard SCD arms (88% vs. 38%, *p* < 0.05) based on pre-treatment dosimetry. Consistent with these findings, this analysis observed a significant difference in the proportion of patients receiving a tumour absorbed dose of ≥ 205 Gy between the MCD and SCD subgroups based on post-treatment dosimetry, with both anatomic segmentation (63% vs. 17%, *p* < 0.05) and count-threshold segmentation (74% vs. 33%, *p* < 0.05).

## Discussion

The compelling results of the DOSISPHERE-01 trial underscored the importance of personalised MCD dosimetry in radioembolization. The present work validated these results by reporting the post-treatment absorbed doses of DOSISPHERE-01 patients and assessing the predictive value of the pre-treatment [^99m^Tc]Tc-MAA absorbed dose distribution. Good correlation was found between pre- and post-treatment absorbed dose metrics with clinically acceptable limits of agreement in Bland-Altman analyses. This data prospectively confirms the predictive accuracy of [^99m^Tc]Tc-MAA based planning in a randomised setting. Furthermore, the dosimetry method used in DOSISPHERE-01 (i.e., count-threshold method) was replicated and compared with the more conventional anatomic method, and the validity of both methodologies confirmed (within the limits of the current sample size).

Pre- and post-treatment dosimetry correlated well. A Pearson correlation coefficient greater than 0.65 was observed for all metrics, except for D95. A suboptimal correlation may be expected for D95 as this metric is highly sensitive to misregistration. These effects disproportionately degraded D95 relative to metrics such as mean dose or D50.

Bland-Altman analysis demonstrated good agreement between pre- and post-treatment dosimetry. For index lesion assessment, the mean difference and limits of agreement observed in this study with anatomic segmentation (11.9 Gy; 95% LoA: − 84.3 to 108.2 Gy) compare favourably with prior reports, including 15.5 Gy (95% CI: − 144 to 175 Gy) by Jadoul et al. [29] and 49.4 Gy (95% CI: 25 to 450 Gy) by d’Abadie et al. [30]. Compared with these studies, mean tumour size was significantly larger in DOSISPHERE-01 and catheter position in the diagnostic and therapeutic angiographies was carefully matched, which may have influenced these results. Consistent with previous publications [17, 30], pre-treatment [^99m^Tc]Tc-MAA measurements tended to overestimate the mean tumour absorbed dose and underestimate the mean normal liver tissue absorbed dose compared with post-treatment ^90^Y dosimetry. However, Bland–Altman analysis demonstrated dispersion for both segmentation methods, and the mean pre- vs. post-treatment absorbed dose differences for anatomic segmentation were not statistically significant.

The DOSISPHERE-01 study utilised a [^99m^Tc]Tc-MAA SPECT count-threshold based segmentation approach, which assumes preferential accumulation of [^99m^Tc]Tc-MAA in the tumour. This approach is unimpeded by co-registration issues. However, its efficacy hinges on the predictive accuracy of the [^99m^Tc]Tc-MAA. Anatomic segmentation, which relies on information derived from diagnostic imaging (CT/MRI) is an easily adopted method for users familiar with response assessment techniques, that exhibits excellent inter-observer reproducibility [31]. However, this method is susceptible to registration errors and potential interval progression. Count-threshold segmentation demonstrated significant pre- vs. post-treatment absorbed dose differences across all metrics and showed greater variability on a case-by-case basis, as reflected in the wider 95% limits of agreement in Bland–Altman plots. Nonetheless, no statistically significant differences were observed between the segmentation methods overall, as indicated by overlapping 95% limits of agreement across all Bland–Altman plots. However, these findings should be interpreted in the context of the limited sample size. Given that both segmentation methods showed good clinical performance, with each method having inherent advantages and limitations, determining the optimal approach requires a case-specific assessment.

Post-treatment dosimetry findings in this post-hoc analysis were also consistent with pre-treatment dosimetry findings of the initial DOSISPHERE-01 trial. Centralised [^99m^Tc]Tc-MAA dosimetry in DOSISPHERE-01 matched post-treatment ^90^Y dosimetry outcomes in the patient subset analysed with count-threshold segmentation (mirroring the methodology used in the original study). Pre-treatment personalised mean tumour absorbed dose reported in DOSISPHERE-01 was 331 Gy (± 132 Gy), which closely aligned with the personalised pre- and post-treatment tumour absorbed dose estimate in this study when a count-threshold segmentation technique was utilised, 336 Gy ± 118 Gy and 273 Gy ± 111 Gy, respectively. Additionally, DOSISPHERE-01 demonstrated a significant difference in the proportion of patients achieving the minimum tumour absorbed dose objective of 205 Gy between the personalised MCD and standard SCD arms, based on [^99m^Tc]Tc-MAA dosimetry. In line with these findings, our analysis confirmed that a significantly higher proportion of MCD patients achieved this threshold compared with SCD patients, based on post-treatment ^90^Y dosimetry. These results validate that the dosimetric differences between personalised MCD and standard SCD strategies, inferred from simulation doses in DOSISPHERE-01, are reproducible when therapeutic doses are directly assessed.

Limitations of this study include the use of two different post-treatment imaging modalities and different imaging protocol parameters, which is known to introduce uncertainties [32] that propagate into the absorbed dose estimates. Additionally, site-specific imaging protocols were not recorded. Dosimetry analysis was blinded to clinical supporting data and cone beam CT images, which impacted the accuracy of contour definition. Cone beam CT is typically used to guide perfused-volume segmentation within the anatomic workflow, the absence of CBCT required reliance on CT/MRI-based vascular anatomy for boundary definition, and in certain cases the [^99m^Tc]Tc-MAA SPECT/CT was used as a secondary adjunct to assist in identifying the edge of the perfused volume. Remaining VOIs were strictly defined using anatomic imaging alone. Contours were not modified for the post-treatment dosimetry evaluation, and due to the time interval between imaging time points, changes in liver volume or morphology may have occurred. Registration between baseline contrast-enhanced CT/MRI imaging and pre- and post-treatment SPECT/PET induces errors in dosimetry, and these effects may increase at larger time intervals. Additionally, SPECT exhibits lower spatial resolution than PET, and the degree of partial volume effect differs between modalities. Accordingly, index lesion segmentations derived from [^99m^Tc]Tc-MAA SPECT count-thresholds are not directly comparable to those obtained from ^90^Y PET imaging. The power of the study was limited by the small population size considered and the mismatch between the patients analysed in DOSISPHERE-01 and those analysed in this ancillary analysis. However, the close alignment in pre-treatment absorbed dose estimates between the separate analyses is noteworthy, suggesting general comparability between the two samples. The relatively high exclusion rate in this study reflects inherent challenges of retrospective, multi-centre data collection. As the DOSISPHERE-01 trial exclusively utilised ^90^Y glass microspheres, caution is warranted when generalising pre- and post-treatment absorbed dose agreement to alternative ^90^Y products.

Further research on the appropriate segmentation method, anatomic, count-threshold or a hybrid [33] of the two, and guidance on the appropriate circumstances for employing a specific method, will help to optimise dosimetry analysis in each clinical scenario. Additionally, greater standardization of imaging protocols and dosimetry methods would be of benefit [34, 35].

## Conclusion

The ancillary analysis of the DOSISPHERE-01 trial reinforces the role of personalised dosimetry in radioembolization, validating that [^99m^Tc]Tc-MAA derived absorbed dose estimates align with post-treatment ^90^Y dosimetry and can readily be used for treatment of HCC patients with ^90^Y glass microspheres radioembolization.

## Supplementary Information

Supplementary Material 1.

## Data Availability

The datasets used and/or analysed during the current study are available from corresponding author on reasonable request.

## Abbreviations

HCC: Hepatocellular Carcinoma
BCLC: Barcelona Clinic Liver Cancer
^90^Y: Ytrrium-90
[^99m^Tc]Tc-MAA: Technetium-99 macroaggregated albumin
SCD: Single compartment dosimetry
MCD: Multi compartment dosimetry
ORR: Objective response rate
SPECT: Single Photon Emission Computed Tomography
DVH: Dose Volume Histogram
OS: Overall survival
PVT: Portal vein thrombosis
PET: Positron emission tomography
MIRD: Medical internal radiation dose
V200: Is the percentage of a volume of interest receiving at least 200 Gy
D95: Dose (Gy) received by at least 95% of the volume of interest
D70: Dose (Gy) received by at least 70% of the volume of interest
D50: Dose (Gy) received by at least 50% of the volume of interest
CT: Computed tomography
MRI: Magnetic resonance imaging
LSF: Lung Shunt Fraction
VOI: Volume of interest
PVNT: Perfused volume normal liver tissue
PCC: Pearsons correlation coefficient
