# Supplementary material for: Predictive value of [99mTc]Tc-MAA SPECT/CT personalised dosimetry in 90Y radioembolization of hepatocellular carcinoma: a DOSISPHERE-01 ancillary study
Source: Eur J Nucl Med Mol Imaging. 2026 Aug 11;53(13):8217–28. doi: 10.1007/s00259-026-08079-8 (PMC13727850; doi:10.1007/s00259-026-08079-8)
Supplement: Supplementary file 1 [file 259_2026_8079_MOESM1_ESM.docx]

**SUPPLEMENTAL TABLES**

**Table 1**. Imaging parameters recommended in the DOSISPHERE-01 trial protocol

| **Modality** | **Parameters** | **^99m^Tc** | **^90^Y** | |
| --- | --- | --- | --- | --- |
| Planar | Window | 140keV ± 7.5% | | 50-150keV |
|  | Matrix size | 256x256 | | 256x256 |
|  | Acquisition time | 5 minutes | | 5 minutes |
|  | Collimator | Low Energy | | Medium Energy |
| Tomo | Spectrometric window | 140keV ± 7.5%  120keV ± 7.5% | | 50-150keV |
|  | Matrix size | 128x128 | | 128x128 |
|  | Acquisition time | 32 proj. of 25 s/head | | 32 proj. of 25 s/head |
|  | Collimator | Low Energy | | Medium Energy |
|  | Algorithm | OSEM 3D | | OSEM 3D |
|  | Subsets | 8 | | 8 |
|  | Iterations | 5 | | 5 |
|  | Scatter correction | Yes | | No |
|  | Attenuation correction | Yes | | Yes* |

NB. No guidance was given on the imaging parameters for the baseline MRI/CT, angiography procedures or the post-treatment ^90^Y PET/CT.

* The DOSISPHERE-01 trial protocol [26] specified that attenuation correction should not be applied to the primary reconstruction in order to preserve access to uncorrected raw data, however, additional reconstructions were permitted. Since attenuation correction was applied to images analysed in the present study, this parameter is reported as "Yes."

**Table 2**. Pre-treatment and post-treatment dose outcomes for index lesion and perfused volume normal liver tissue, in the personalised (MCD) and standard (SCD) subgroups

| **Segmentation method** | **Personalised (MCD) or standard (SCD)** | **Structure** | **Pre-treatment**  **mean dose**  **(SD)**  **(Gy)** | **Post-treatment**  **mean dose (SD)**  **(Gy)** | **P-value** |
| --- | --- | --- | --- | --- | --- |
| **Anatomic** | **MCD** | Index lesion  PVNT | 270 (106)  98 (43) | 256 (103)  114 (40) | 0.32  0.09 |
|  | **SCD** | Index lesion  PVNT | 211 (130)  83 (56) | 201 (134)  83 (49) | 0.29  0.98 |
| **Count Threshold** | **MCD** | Index lesion  PVNT | 336 (118)  74 (38) | 273 (111)  114 (44) | <0.05  <0.05 |
|  | **SCD** | Index lesion  PVNT | 256 (150)  66 (46) | 218 (138)  79 (44) | <0.05  <0.05 |

**Table 3**. Pre- and post-treatment dosimetry statistics for n=42 patients (the data includes 5 additional patients excluded from the primary analysis due to absence of a baseline (CT/MRI) dataset) using count-threshold segmentation

| **Structure** | **Pre-Treatment**  **Mean Dose**  **(SD)**  **(Gy)** | **Post-Treatment**  **Mean Dose**  **(SD)**  **(Gy)** | **Dose Difference**  **(95% LoA)**  **(Gy)** | **P-value** |
| --- | --- | --- | --- | --- |
| Tumour | 284 (138) | 234 (125) | 49.4 (-232.8, 331.7) | 0.03 |
| PVNT | 65 (41) | 93 (47) | -27.8 (-133.1, 77.5) | <0.05 |

**SUPPLEMENTAL FIGURES**

**Figure 1**. Bland-Altman plots for the index lesion dose volume coverage analysis. The difference between the dose to 50% of the index lesion (D50) for [^99m^Tc]Tc-MAA and ^90^Y is plotted against post-treatment index lesion D50 for (A) anatomic segmentation and (B) count-threshold based segmentation. The 95% limits of agreement (LoA) are indicated and surrounded by two dotted lines indicating the standard error of the 95% LoA

13.7

111.9

-84.4

47.4

168.7

-74.0

**A**

**B**

**Figure 2**. Bland-Altman plots for the index lesion dose volume coverage analysis. The difference between the dose to 70% of the index lesion (D70) for [^99m^Tc]Tc-MAA and ^90^Y is plotted against post-treatment index lesion D70 for (A) anatomic segmentation and (B) count-threshold based segmentation. The 95% limits of agreement (LoA) are indicated and surrounded by two dotted lines indicating the standard error of the 95% LoA

**B**

**A**

8.8

121.7

-104.0

55.0

189.0

-79.0

**Figure 3**. Bland-Altman plots for the index lesion dose volume coverage analysis. The difference between the dose to 95% of the index lesion (D95) for [^99m^Tc]Tc-MAA and ^90^Y is plotted against post-treatment index lesion D95 for (A) anatomic segmentation and (B) count-threshold based segmentation. The 95% limits of agreement (LoA) are indicated and surrounded by two dotted lines indicating the standard error of the 95% LoA

**B**

**A**

-8.6

104.6

-121.7

86.1

259.2

-87.1

**Figure 4**. Bland-Altman plots for the index lesion dose volume coverage analysis. The difference between the volume of the index lesion receiving 200Gy (V200) for [^99m^Tc]Tc-MAA and ^90^Y is plotted against post-treatment index lesion V200 for (A) anatomic segmentation and (B) count-threshold based segmentation. The 95% limits of agreement (LoA) are indicated and surrounded by two dotted lines indicating the standard error of the 95% LoA

**B**

**A**

4.2

39.8

-31.3

13.9

60.1

-32.3

**Figure 5**. Example of disagreement between pre- and post-treatment index lesion absorbed dose. (A) (C), Pre-treatment [^99m^Tc]Tc-MAA SPECT/CT images. (B) (D) Post-treatment ^90^Y PET/CT images. The predicted pre-treatment absorbed dose was 168 Gy, compared with a post-treatment absorbed dose of 120 Gy, indicating overestimation by [^99m^Tc]Tc-MAA SPECT/CT-based dosimetry. In this case the difference in dose distribution is attributed to the differing uptake of the [^99m^Tc]Tc-MAA scout agent and ^90^Y microspheres. (E), Dose–volume histogram curves of the index lesion VOIs from the pre- and post-treatment dosimetry assessments.

**B**

**A**

**D**

**C**

**E**

**Figure 6**. Bland-Altman plots for the index lesion dose for n=42 patients (the data includes 5 additional patients excluded from the primary analysis due to absence of a baseline (CT/MRI) dataset). The difference between the index lesion dose for [^99m^Tc]Tc-MAA and ^90^Y is plotted against post-treatment index lesion dose for count-threshold based segmentation. The 95% limits of agreement (LoA) are indicated and surrounded by two dotted lines indicating the standard error of the 95% LoA

49.4

331.7

-232.8

**Figure 7**. Bland-Altman plots for the PVNT dose for n=42 patient (the data includes 5 additional patients excluded from the primary analysis due to absence of a baseline (CT/MRI) dataset). The difference between the PVNT dose for [99mTc]Tc-MAA and 90Y is plotted against post-treatment PVNT dose for count-threshold based segmentation. The 95% limits of agreement (LoA) are indicated and surrounded by two dotted lines indicating the standard error of the 95% LoA

77.5

-27.8

-133.1
